# Rectifying Performance of Heterojunction Based on *α*-Borophene Nanoribbons with Edge Passivation

**DOI:** 10.1186/s11671-020-03417-7

**Published:** 2020-09-24

**Authors:** Guoliang Yu, Wence Ding, Xianbo Xiao, Xiaobo Li, Guanghui Zhou

**Affiliations:** 1 Department of Physics, Key Laboratory for Low-Dimensional Structures and Quantum Manipulation (Ministry of Education), Hunan Normal University, Changsha, 410081 China; 2 School of Computer Science, Jiangxi University of Traditional Chinese Medicine, Nanchang, 330004 China; 3 School of Mathematics and Statistics, Hunan University of Technology and Business, Changsha, 410215 China

**Keywords:** *α*-Borophene nanoribbon, Lateral heterojunction, Rectification effect, First-principles calculation

## Abstract

We propose a planar model heterojunction based on *α*-borophene nanoribbons and study its electronic transport properties. We respectively consider three types of heterojunctions. Each type consists of two zigzag-edge *α*-borophene nanoribbons (Z *α*BNR), one is metallic with unpassivated or passivated edges by a hydrogen atom (1H-Z *α*BNR) and the other is semiconducting with the edge passivated by two hydrogen atoms (2H-Z *α*BNR) or a single nitrogen atom (N-Z *α*BNR). Using the first-principles calculations combined with the nonequilibrium Green’s function, we observe that the rectifying performance depends strongly on the atomic structural details of a junction. Specifically, the rectification ratio of the junction is almost unchanged when its left metallic ribbon changes from ZBNR to 1H-Z *α*BNR. However, its ratio increases from 120 to 240 when the right semiconducting one varies from 2H-Z *α*BNR to N-Z *α*BNR. This rectification effect can be explained microscopically by the matching degree the electronic bands between two parts of a junction. Our findings imply that the borophene-based heterojunctions may have potential applications in rectification nano-devices.

## Introduction

Over the past decades, a great number of two-dimensional (2D) materials, including graphene [1, 2], silicene [3, 4], transition metal dichalcogenides (TMD) [5, 6], and phosphorene [7, 8], have been extensively studied due to their unique properties. Especially, these 2D materials demonstrate some interesting electronic transport behaviors, such as giant magneto resistance (GMR) [9, 10], negative differential resistance (NDR) [11, 12], spin filtering [13, 14], and rectification [15, 16], thus having potential applications in nanoscale electronic devices. Recently, some studies have also shown that 2D materials have broad application prospects in nanoscale thermoelectric devices [17–20]. Subsequently, the research on lateral heterojunctions based on 2D materials becomes an important topic. And some theoretical studies have showed that the lateral heterojunctions have potential applications in field effect transistor and complementary metal oxide semiconductor technologies [21, 22]. Further, the lateral heterojunctions with atomic thickness have already been prepared in experiments [23, 24]. These achievements have inspired the effort for further exploring lateral heterojunctions made of more suitable 2D materials.

Recently, borophene monolayers have also received extensive interests [25–28] after graphene and silicene. The theoretical studies predicted that the monolayer boron sheets can be stably existed on the metallic substrate, which was confirmed by the subsequent observations [29, 30]. So far, a number of 2D boron structures have been obtained by epitaxial growth on Ag (111) substrates, such as *β*_12_-, *χ*_3_-, *δ*_6_-borophene and honeycomb borophene [31–34]. Theoretical studies point out that the stability of the boron sheet can be increased by introducing a hexagonal hole [35]. The DFT calculations indicated that the borophene with a “hexagon hole density” (*η*) of 1/9, named as *α*−borophene [35, 36], is favorable in terms of energy. Further, the zigzag edge *α*-borophene nanoribbon (Z *α*BNR) exhibits either metallic or semiconducting behavior through different edge modifications [37]. Hence, the electronic transport property for borophene nanostructures remains to be explored further, although a large number of studies have been carried out on the electronic structures, mechanical and thermal properties [25–28].

In this work, we investigate the transport properties of heterojunctions made of the zigzag edge Z *α*BNRs. We construct three types of in-plane metal-semiconductor lateral junctions. We find that all the junctions exhibit rectification behavior in the low bias regime due to the presence of the interfaces in the scattering region and the asymmetry on the left and right sides. Moreover, the rectifying effect of the junctions becomes pronounced with the increase of primitive cell numbers in the semiconductor part of the junction. The transport properties of junctions strongly depended on right part semiconducting nanoribbons. This phenomenon can be ascribed to the band gap near the Fermi level of the semiconducting part. The probability of electrons through the junction to be smaller when the band gap is increasing, which causes the current of the junction decreased and the rectification ratio increased. In particular, the rectification rate of junction M10N can reach about 240, which is comparable to the previously studied heterojunction with graphene as an electrode and indicates that it has potential applications in rectification devices [38]. The organization of this paper is as follows. In the “Model and Computational Methods” section, we describe the computational details. In the “Results and Discussion” section, we present the transport properties of the proposed junctions. Finally, we summarize our results in the “Conclusions” section.

## Model and Computational Methods

**Fig. 1:**
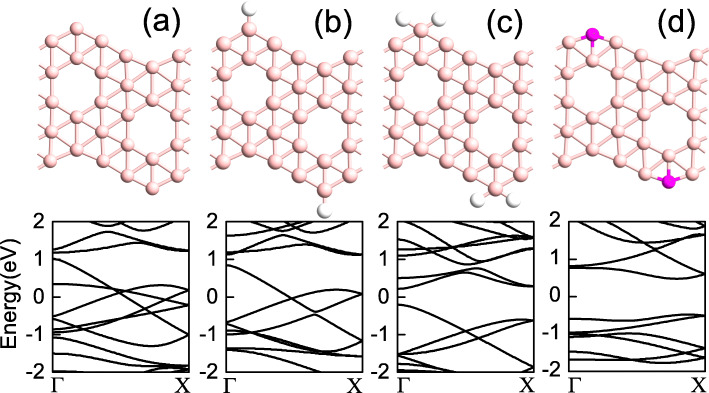
The unit cell geometries (upper) and band structures (lower) for **a** unpassivated Z *α*BNR, **b** 1H-Z *α*BNR, **c** 2H-Z *α*BNR, and **d** N-Z *α*BNR, where the Fermi level is set to zero, and the pink, magenta, and white spheres represent boron, nitrogen, and hydrogen atoms, respectively

The unit cells of the considered Z *α*BNRs without or with outmost edge-apex modifications are shown in the upper part of Fig. 1, where (a) for the unpassivated Z *α*BNR, (b–d) for the Z *α*BNRs with the outmost edge boron atoms of the cell passivated by one hydrogen (H), two H atoms and replaced by a nitrogen (N) atom, which are named as 1H-Z *α*BNR, 2H-Z *α*BNR, and N-Z *α*BNR, respectively. And their corresponding electronic energy dispersions are subsequently shown in the lower part of Fig. 1, from which we can identify the difference in band structure for the ribbons. From Fig. 1a, several bands of the intrinsic pristine Z *α*BNR crosses over the Fermi level (*E*_*F*_), which exhibits metallic property. For 1H-Z *α*BNR, since the partially dangling bonds are saturated with H atoms, the number of bands near the *E*_*F*_ are less than those for the unpassivated one and also exhibits metal behavior. For 2H-Z *α*BNR, however, the *E*_*F*_ moves to the gap between the bonding and antibonding bands due to the dangling bonds at the edge are saturated with two H atoms. Therefore, 2H-Z *α*BNR is a semiconductor with a 0.43 eV direct band gap at the *Γ*-point as shown in Fig. 1c. We mention that our results of band structure for H-passivated ribbons here agree well with the previous numerical calculations [37]. Moreover, as shown in Fig. 1d, the band structure of N-Z *α*BNR indicates that it is a semiconductor with a 1.0 eV indirect band gap. This may be owing to the substitution of N to the B atomic positions at the edge, which brings enough electrons to fill the bonding orbits.

**Fig. 2:**
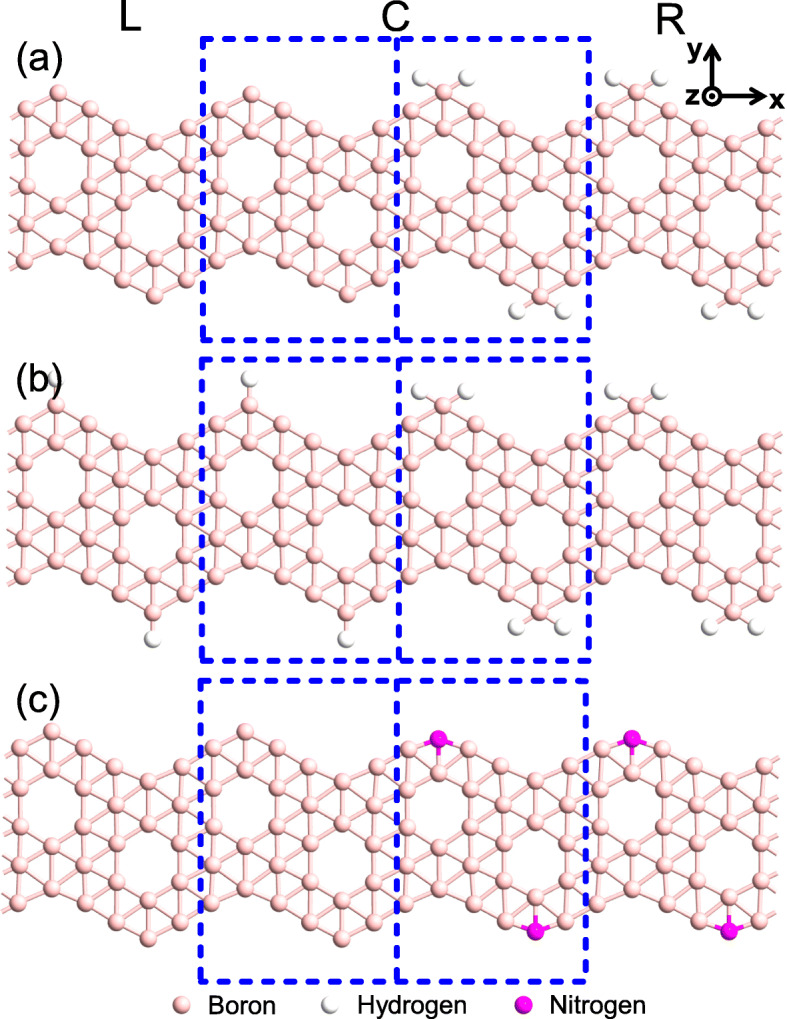
The geometry structures of the proposed three types of model junctions, where **a** for M*n*H, **b** for M’*n*H, and **c** for M*n*N, in which *n* represents the number of unit cells of the semiconductor part in the central scattering. The large (blue) dashed frame represents the central scattering region in which the small one indicates the unit cell

We establish three metal/semiconductor lateral heterojunction models based on the above mentioned Z *α*BNRs. Each model junction is divided into three parts: the left electrode, the right electrode, and the central scattering region. The geometry structure of the junctions, as shown in Fig. 2, where the left electrode is always a semi-infinitive long bare unpassivated Z *α*BNR or 1H- Z *α*BNR, and the right electrode is either a semiconducting 2H- or N-Z *α*BNR. Particularly, however, the central scattering regions of the three junctions are a Z *α*BNR unit cell coupled with *n* (*n* = 1, 2, 5, 8, 10) unit cells of 2H-Z *α*BNR, a 1H-Z *α*BNR coupled with *n* cells of 2H-Z *α*BNR, and a Z *α*BNR cell coupled with *n* cells of N-Z *α*BNR, respectively. Likewise, we accordingly name them as the M*n*H, M’*n*H and M*n*N junctions, which are shown in Fig. 2a–c, respectively. It is worth noting that Fig. 2 only shows a schematic diagram of the model with *n* = 1 and the other cases of *n* are omitted for saving the space.

The calculations were performed using the software package Atomistix ToolKit (ATK), QuantumWise A /S (www.quantumwise.com), which is based on the DFT combined with the Keldysh nonequilibrium Green’s function (NEGF) [39–41]. The Perdew-Burke-Ernzerhof (PBE) functional under the generalized gradient approximation (GGA) is used for the exchange-correlation potential. The Borilliouin zone (BZ) is sampled by using a 1×1×100 Monkhorst-Pack *k*-mesh, and the cutoff energy is set to 150 Ry. The geometric structures of all heterojunctions were relaxed until the absolute value of force acting on each atom is less than 0.01 eV Å ^−1^. In order to avoid interactions between periodic images, the supercell at least has a vacuum layer thickness of 15 Å.

The current through the heterojunction under a bias voltage *V* is calculated by the Landauer-Büttiker formula [42, 43]

$$I(V)=2e/h\int{T(E,V)\left[f_{L}(E,V)-f_{R}(E,V)\right]dE}, $$

where *h*, *e*, and *V* are respectively the Planck’s constant, the elementary charge, and the bias voltage, and *f*_*L*/*R*_(*E*,*V*) is the Fermi-Dirac distribution function in the left/right electrode. The transmission coefficient is calculated by

$$T(E,V)=Tr\left[\Gamma_{L}(E,V) G(E,V)\Gamma_{R}(E,V) G^{\dag}(E,V)\right], $$

where *G*(*E*,*V*) and *G*^*†*^(*E*,*V*) denote the retarded and advanced Green’s function, respectively, and *Γ*_*L*_ (*Γ*_*R*_) is the coupling matrix between the central scattering region with the left (right) electrode.

## Results and Discussion

**Fig. 3:**
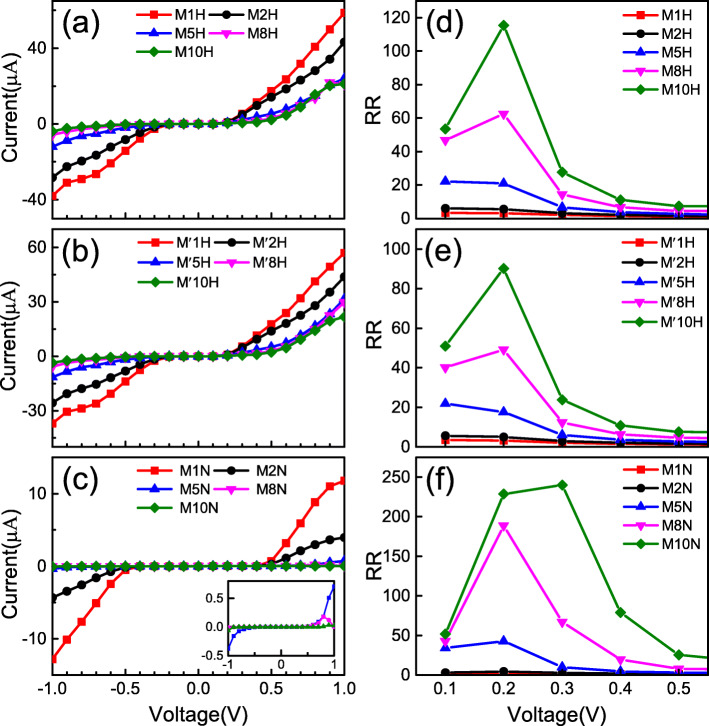
The *I*-*V* characteristics and rectification ratios for the three types of heterojunctions, where **a**–**c** correspond to *I*-*V* curves for junctions M*n*H, M ^′^*n*H, and M*n*N (*n* = 1, 2, 5, 8, 10) within bias range of (− 1,1) V, respectively. The inset in **c** is the enlarged *I*-*V* curves of M*n*N within the bias range. **d**–**f** The rectification ratios calculated correspondingly from the *I-V* data

The calculated current −voltage (*I*−*V*) curves of heterojunctions M*n*H, M ^′^*n*H, and M*n*N within the bias range of −1.0 to 1.0 V are shown in Fig. 3a–c, respectively. From these *I*−*V* curves, we can clearly see that with the increment of positive bias, the current increases rapidly in all three types of junctions. However, with the increase of the negative bias, the current through the junctions are increased more slowly. The *I*−*V* curves have obviously asymmetric characteristics under the whole bias, which means that the junctions have a rectification behavior within the bias range. The rectification effect in the heterojunction is mainly caused by the asymmetry of the different nanoribbons on the left and right sides and the formation of the interface in the central scattering region. In order to evaluate the strength of the rectification behavior, we use the data for the *I*−*V* curves to calculate the rectification ratio (RR), which is defined as RR (*V*)= |*I*(+*V*)|/|*I*(−*V*)|, where *I*(±*V*) represents the current under positive and negative bias. The calculated RRs of the three types of junctions M*n*H, M ^′^*n*H, and M*n*N within the 0.1 V −0.5 V bias range are shown in Fig. 3d–f, respectively. For type M*n*H, the RR of M1H is only 3 at 0.2 V while that of M10H can reach 115 at the same bias. Similarly, for the M ^′^*n*N type at bias 0.2 V, the RR of M ^′^1H is 3 and that of M ^′^10H is up to 90. Moreover, for the M*n*N type, the RR of M1N is 2 at 0.3 V while that of M10N reaches up to 240. Further, by careful observation on Fig. 3, we find that the magnitude of the current and RR can be controlled by changing the size of the semiconductor part of the junction. In specific, on the one hand, the current in the junction is reduced with the number of primitive cells of the semiconductor part is increased. On the other hand, the RR is significantly increased with the number of primitive cells is increased. Since the right side of the heterojunction is a semiconductor nanoribbon with a band gap, the probability of electron tunneling decays exponentially as the length of the semiconductor increases. As a result, in the heterojunctions of M*n*H, M ^′^*n*H, and M*n*N, as *n* increases, RR increases significantly. This result is in good agreement with previous studies on the heterojunctions based on other 2D materials [44–46].

**Fig. 4:**
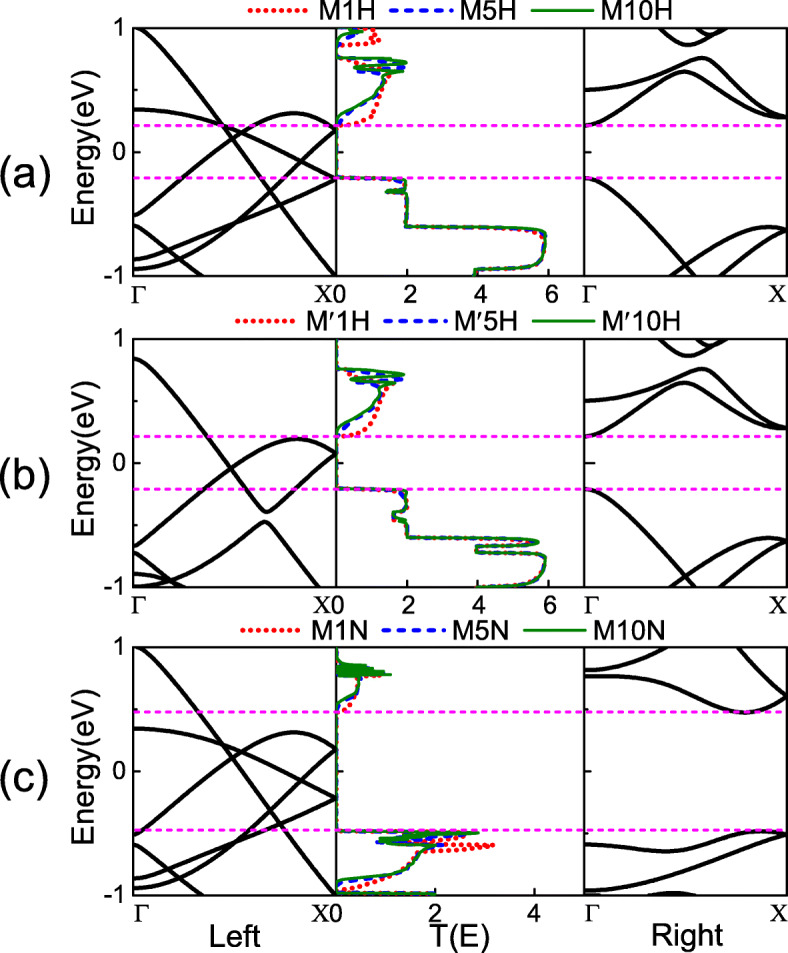
The band structure of the left and right electrode, where the Fermi level is set to zero and the magenta dashed lines indicate the band gap of the right semiconductor electrode. The transmission spectra at zero bias for heterojunctions **a** M*n*H, **b** M ^′^*n*H, and **c** M*n*N with *n*=1 (red dashed line), 5 (blue dashed line), and 10 (green solid line) are correspondingly shown in the middle part of each figures, respectively

Comparing the *I*−*V* curves and RRs among the three types of heterojunctions shown in Fig. 3, we find that the variation of *I*−*V* curves and RRs for M*n*H and M ^′^*n*H have the similar trends. However, those for M*n*N are significantly different. In order to explain the difference in transport properties of the three types of junctions, we have calculated the transmission spectra under zero bias shown in Fig. 4, where the band structures of the left and right electrode are accompanied. From these transmission spectra, one can see that all of the junctions have a transmission gap near the Fermi level, where we use the magenta dotted line to denote the gap position. The reason for the existence of the transmission gap is that the energy band structure of the right electrode has a gap near the Fermi level. Thus, the band structure of the left and right electrodes does not match, causing the transport channel to be closed, and the electrons of the left electrode cannot reach the right electrode. This is also the physical origin of the weak current at the low bias. Additionally, the comparison of Fig. 4a, b and Fig. 4a, c shown that the transmission spectra of M*n*H and M ^′^*n*H under zero bias has similar trends; however, the trends of M*n*H and M*n*N are quite different. This is determined by the matching degree of the left and right electrodes band structures near the Fermi level. The left metallic nanoribbon of the junction M ^′^*n*H changes from Z *α*BNR to 1H-Z *α*BNR compared to M*n*H. The matching degree between left and right electrodes near the Fermi level is almost unchanged. However, for M*n*N, the right semiconductor nanoribbon is changed from 2H-Z *α*BNR to N-Z *α*BNR compared to M*n*H. The band gap is increased from 0.43 eV to 1.0 eV, which results in a decrease in the matching degree of the left and right electrodes near the Fermi level. Therefore, the transport properties of M*n*H and M ^′^*n*H are almost same, while the M*n*H and M*n*N are obviously different. This result indicates that changing the left part metallic nanoribbon has a little effect on the transport properties of the junction; however, changing the right part semiconductor nanoribbon has a great influence on it.

**Fig. 5:**
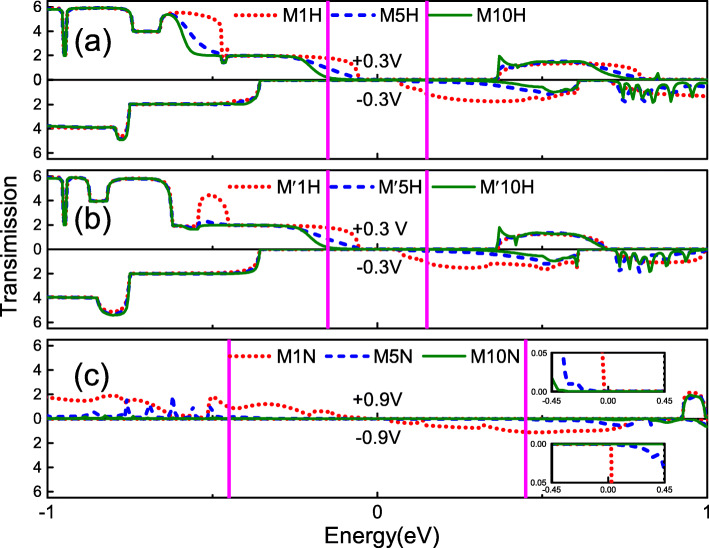
The transmission spectra for heterojunctions **a** M*n*H at a bias ± 0.3 V, **b** M ^′^*n*H at a bias ± 0.3 V, and **c** M*n*N at a bias ± 0.9 V with the same choice of *n* in line colors for Fig. 4, where in each figure the upper/lower part for the transmission at the positive/negative bias. The two vertical (magenta) solid lines indicate the bias window. The inset in Fig. 5c is an amplification of the transmission spectra in the bias window

To further understand the details of the rectification behavior for the heterojunctions, we calculated the transmission spectra at several certain biases, as shown in Fig. 5, where the above/below part shows the transmission spectra of the junction under the positive/negative bias. According to the Landauer-Büttiker formula, we know that the current in the junction is directly related to the integrated area of the transmission spectrum within the bias window [47–49]. From the transmission spectrum shown in Fig. 5, we can see that the three types of models have a common trend. In the bias window, the integrated area of the transmission spectrum decreases with the number of primitive cells in the semiconductor part is increased. This is why the current in the heterojunction decreases with the number of cells in the semiconductor portion is increased, as shown in Fig. 3. Figure 5a shows the transmission spectra of the heterojunctions M*n*H at ± 0.3 V. For M1H, the integral area of the transmission spectrum in the bias window at 0.3 V is only slightly greater than − 0.3 V. Hence, the current of 0.3 V is only slightly higher than − 0.3 V, and the RR is only 3 at the bias 0.3 V. However, for M5H and M10H, the integral area of the transmission spectrum under positive bias in the bias window is significantly greater than under negative bias. This leads to the current of the M5H and M10H under positive bias being greater than under negative bias, and the RR is much larger than M1H. Figure 5b shows the transmission spectra of M ^′^*n*H at ± 0.3 V. From the figure, one can see that the transmission spectra of M ^′^*n*H in the bias window are almost the same as M*n*H. Therefore, under the same bias voltage, the current and the RR of M ^′^*n*H and M*n*H are nearly the same [see Fig. 3b, e]. The transmission spectra of M*n*N at ±0.9 V are shown in Fig. 5c. Since the transmission coefficients in the bias window are too small, we magnify the transmission spectra in the bias window and attach it as an inset to the lower right side of Fig. 5c. The trend of the M1N transmission spectrum in the bias window is similar to the M1H and M ^′^1H. Therefore, the RR of M1N is also small. For M5N and M10N, the integral area of the transmission spectrum under positive bias in the bias window is much larger than the area under negative bias. Therefore, compared with M1N, the asymmetric characteristics of these *I*−*V* curves are more obvious. This implies that they have a large rectification ratio. It is worth mentioning that the RR of M10N can reach 240, which is the best among the three types heterojunction.

**Fig. 6:**
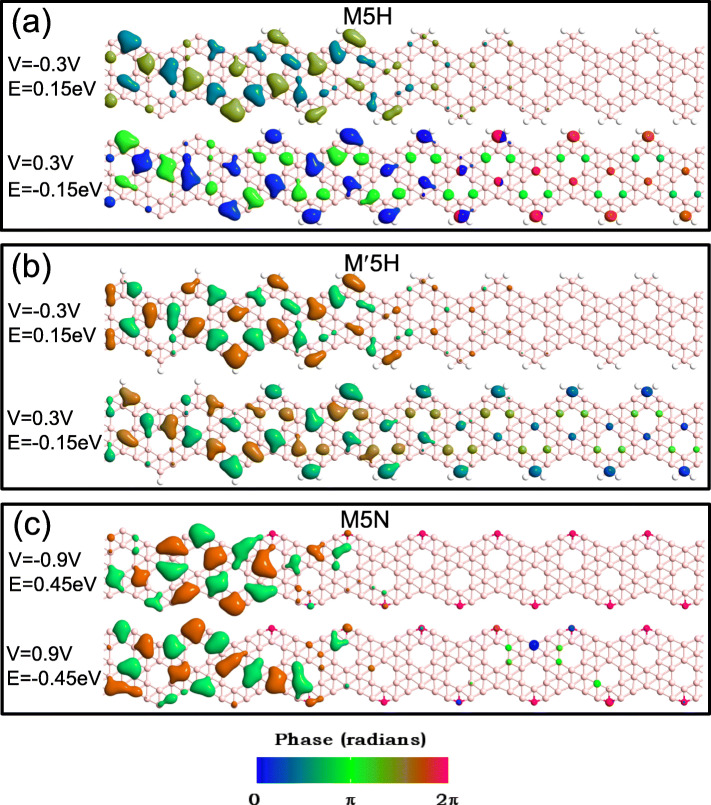
The transmission eigenstates for heterojunction **a** M5H at bias − 0.3 V with *E*=0.15 eV (upper) and at bias 0.3 V with *E*= − 0.15 eV (lower), **b** M ^′^5H at − 0.3 V with *E*=0.15 V and at 0.3 V with *E*= − 0.15 eV, and **c** M5N at bias= − 0.9 V with *E*=0.45 eV and at 0.9 V with *E*= − 0.45 eV, respectively. The isovalues are fixed at 0.2 Å ^−3^*eV*^−1^ for all eigenstates

In order to more intuitively explain the transmission spectrum in Fig. 5, we show the transmission eigenstate of M5H and M ^′^5H at *V* = 0.3 V, *E* = − 0.15 eV, and *V* = − 0.3 V, *E* = 0.15 eV in Fig. 6a and b, respectively. And the transmission eigenstate of M5N at *V* = 0.9 V, *E* = − 0.45 eV, and *V* = − 0.9 V, *E* = 0.45 eV are shown in Fig. 6c [15, 16, 49]. The analysis of transmission eigenstate can obtained by linearly combining the propagating Bloch states $\sum _{m}C_{a,m}\psi _{m}$. The *C*_*a*,*m*_ can be derived from the diagonalization of the transmission matrix, i.e., ${\sum \nolimits }_{n}T_{mn}C_{a,n}$= *λ*_*a*_*C*_*a*,*m*_, where *λ*_*a*_ is the transmission eigenvalue. As can be seen from Fig. 6, for all heterojunctions, the transmission eigenstate under negative bias is located in the metallic part (unpassivated Z *α*BNR and 1H-Z *α*BNR). At positive bias, the transmission eigenstate is mostly localized on the left part. However, it forms a transmission channel in the heterojunction. The electrons can be transferred from the left electrode to the right electrode. Therefore, in the bias window, the transmission coefficient under positive bias is greater than the under negative bias. In comparison Fig. 6a with b, one can see that the transmission eigenstate of M ^′^5H and M5H are only slightly different. Thus, the heterojunctions M ^′^5H and M5H have almost the same transmission coefficients in the bias window. In addition, for M5N, since the band gap of the semiconductor part increases, which results in more dramatic electronic scatter in the heterojunction. Therefore, only a few of the transmission eigenstates can be transmitted to the right side. This led to the transmission coefficient of M*n*N in the bias window is smaller than that of the other two types of heterojunction. Meanwhile, at the same bias, the current of M*n*N is the smallest of the three types of heterojunctions.

**Fig. 7:**
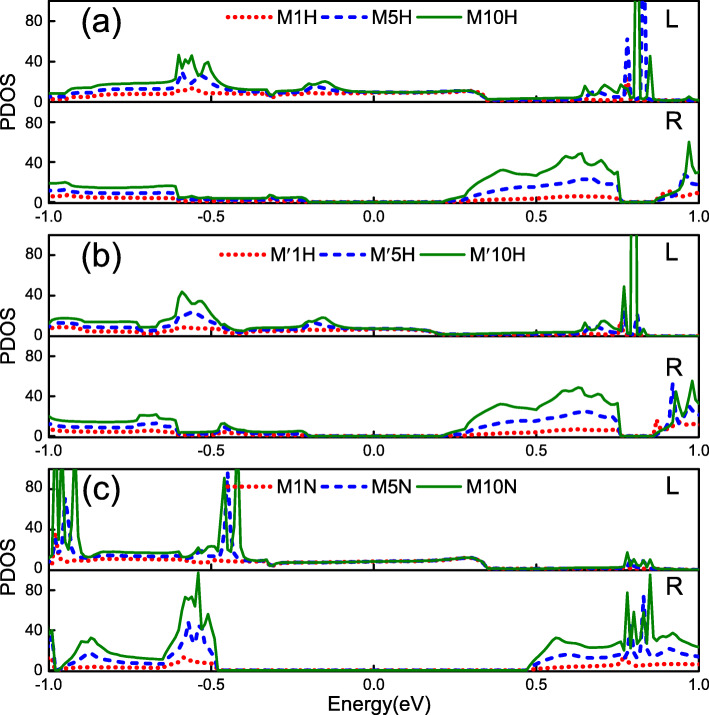
The projected density of states (PDOS) of the left unpassivated ZBNR electrode (upper) and the right electrode (1H-Z *α*BNR, 2H −ZBNR, or N-Z *α*BNR) (lower) for **a** M*n*H, **b** M ^′^*n*H, and **c** M*n*N with the same choice of *n* in line colors for Fig. 5, respectively

Finally, in order to further explore the effect of the left and right nanoribbons on the transport properties with heterojunctions, Fig. 7 shows the projected density of states (PDOS) of the three types of heterojunctions. From Fig. 7a, one can see that the PDOS spectra contributed by the left electrodes (unpassivated Z *α*BNR) of the junctions M1H, M5H, and M10H with overlap together near the Fermi level. This indicates that the PDOS contributed by the left electrode is hardly affected by the extension of the semiconductor nanoribbon (2H-Z *α*BNR) in the center scattering region. However, the PDOS spectra contributed by the right electrode (2H-Z *α*BNR) has a gap near the Fermi level. This is caused by a band gap near the Fermi level of the right electrode [see Fig. 3c]. Affected by the extension of the intermediate scattering region 2H-Z *α*BNR, the PDOS spectra contributed by the right electrodes of junctions M1H, M5H, and M10H differ greatly from each other in the energy range outside the band gap. Since there are no essential difference between the two electrodes for heterojunction M ^′^*n*H and M*n*H, the right electrode is the same and the left electrode is metallic ribbon. So, the PDOS of M ^′^*n*H and M*n*H are almost the same near the Fermi level, as shown in Fig. 7a, b. This is one of the reasons why the transmission spectrum, *I*−*V* curves and RR of M*n*H and M ^′^*n*H are similar under low bias [see Figs. 3 and 5]. In Fig. 7c, we present the PDOS of the M*n*N. Due to the band gap of the semiconductor part in the heterojunction increases, the effect of the left electrode on transmission properties becomes smaller. Therefore, PDOS overlaps each other within a larger energy range near the Fermi level. The PDOS spectrum contributed by the right electrode exists a gap in the energy range of (− 0.5, 0.5) eV. They are consistent with the position of the gap with N −ZBNR band structure. From the PDOS, we can conclude that the left side metal electrode has little effect on the transport properties of the intermediate scattering region. However, the semiconductor part electrode on the right is critical to the transport properties of the intermediate scattering region.

## Conclusions

In summary, we have studied the transport properties of *α*−borophene based three type heterojunctions. We found that the three types of heterojunctions exhibit rectification behavior, among which the rectification ratio of heterojunction Z *α*BNR/N-Z *α*BNR can reach up to 240. Moreover, as the number of unit cells in the central semiconductor part increases, the effect of rectification becomes more obvious. The origin of the rectification behavior is revealed and discussed by analyzing the transmission spectra and eigenstates under positive/negative bias. The rectification behavior of the heterojunctions strongly depends on the band gap value of the nanoribbons in the semiconductor part. This conclusion was further confirmed by analyzing PDOS contributed by the left and right electrodes. Our results provide new lines for the design of rectifying electronic devices.

## Data Availability

The design of nanojunctions and computational calculations were carried out by ATK.

## Abbreviations

2D: Two-dimensional
TMD: Transition metal dichalcogenides
GMR: Giant magneto resistance
NDR: Negative differential resistance
DFT: Density functional theory
Z *α*BNR: Zigzag-edge *α*-borophene nanoribbons
H: Hydrogen atom
N: Nitrogen atom
1H-Z *α*BNR: The Z *α*BNRs with the edge passivated by one hydrogen
2H-Z *α*BNR: The Z *αBNRs with the edge passivated by two hydrogen atoms*
N-Z *α*BNR: The Z *α*BNRs with the edge boron atoms replaced by a nitrogen atom
*E*_*F*_: Fermi level
ATK: Atomistix toolKit
NEGF: The Keldysh nonequilibrium Green’s function
PBE: Perdew-Burke-Ernzerhof
GGA: Generalized gradient approximation
BZ: Borilliouin zone
*I*−*V* curves: Current −voltage curves
RR: Rectification ratio
PDOS: Projected density of states
